# Therapeutic and Prognostic Relevance of Cancer Stem Cell Populations in Endometrial Cancer: A Narrative Review

**DOI:** 10.3390/diagnostics15151872

**Published:** 2025-07-25

**Authors:** Ioana Cristina Rotar, Elena Bernad, Liviu Moraru, Viviana Ivan, Adrian Apostol, Sandor Ianos Bernad, Daniel Muresan, Melinda-Ildiko Mitranovici

**Affiliations:** 1 Obstetrics and Gynecology I, Mother and Child Department, “Iuliu Hatieganu” University of Medicine and Pharmacy, 400012 Cluj-Napoca, Romania; cristina.rotar@umfcluj.ro (I.C.R.); muresandaniel01@elearn.umfcluj.ro (D.M.); 2 Department of Obstetrics and Gynecology, Center for Neuropsychology and Behavioral Medicine, Center for Laparoscopy, Laparoscopic Surgery and In Vitro Fertilization, “Victor Babes” University of Medicine and Pharmacy from Timișoara, 300041 Timișoara, Romania; 3 Ist Clinic of Obstetrics and Gynecology, Laparoscopy, In Vitro Fertilization and Embryotransfer Research Center, “Pius Brinzeu” County Clinical Emergency Hospital, 300723 Timișoara, Romania; 4 Department of Anatomy, University of Medicine, Pharmacy, Sciences and Technology “George Emil Palade”, 540142 Targu Mures, Romania; liviu.moraru@umfst.ro (L.M.); mitranovicimelinda@yahoo.ro (M.-I.M.); 5 Department of Cardiology, Victor Babes” University of Medicine and Pharmacy, 2 Eftimie Murgu Sq, 300041 Timisoara, Romania; ivan.viviana@umft.ro (V.I.); adrian.apostol@umft.ro (A.A.); 6 Centre for Fundamental and Advanced Technical Research, Romanian Academy—Timisoara Branch, No 24 Mihai Viteazul Boulevard, 300223 Timisoara, Romania; sandor.bernad@upt.ro; 7 Research Centre for Engineering of Systems with Complex Fluids, Politehnica University Timisoara, No 1 Mihai Viteazul Boulevard 1, 300222 Timisoara, Romania

**Keywords:** endometrial cancer, stemness, prognostic factors, targeted treatment, stemness markers, stemness pathway

## Abstract

The biggest challenge in cancer therapy is tumor resistance to the classical approach. Thus, research interest has shifted toward the cancer stem cell population (CSC). CSCs are a small subpopulation of cancer cells within tumors with self-renewal, differentiation, and metastasis/malignant potential. They are involved in tumor initiation and development, metastasis, and recurrence. Method. A narrative review of significant scientific publications related to the topic and its applicability in endometrial cancer (EC) was performed with the aim of identifying current knowledge about the identification of CSC populations in endometrial cancer, their biological significance, prognostic impact, and therapeutic targeting. Results: Therapy against the tumor population alone has no or negligible effect on CSCs. CSCs, due to their stemness and therapeutic resistance, cause tumor relapse. They target CSCs that may lead to noticeable persistent tumoral regression. Also, they can be used as a predictive marker for poor prognosis. Reverse transcription–polymerase chain reaction (RT-PCR) demonstrated that the cultured cells strongly expressed stemness-related genes, such as SOX-2 (sex-determining region Y-box 2), NANOG (Nanog homeobox), and Oct 4 (octamer-binding protein 4). The expression of surface markers CD133+ and CD44+ was found on CSC as stemness markers. Along with surface markers, transcription factors such as NF-kB, HIF-1a, and b-catenin were also considered therapeutic targets. Hypoxia is another vital feature of the tumor environment and aids in the maintenance of the stemness of CSCs. This involves the hypoxic activation of the WNT/b-catenin pathway, which promotes tumor survival and metastasis. Specific antibodies have been investigated against CSC markers; for example, anti-CD44 antibodies have been demonstrated to have potential against different CSCs in preclinical investigations. Anti-CD-133 antibodies have also been developed. Targeting the CSC microenvironment is a possible drug target for CSCs. Focusing on stemness-related genes, such as the transcription pluripotency factors SOX2, NANOG, and OCT4, is another therapeutic option. Conclusions: Stemness surface and gene markers can be potential prognostic biomarkers and management approaches for cases with drug-resistant endometrial cancers.

## 1. Introduction

Endometrial cancer (EC) is a malignant disease that originates in the endometrium, and it is the most common gynecological malignancy in developed countries [1,2]. According to the latest available data from the European Cancer Information System (ECIS) and the World Health Organization’s International Agency for Research on Cancer (IARC), the incidence of EC has increased due to improvements in cancer diagnosis and also due to changes in hormonal factors [3,4,5]. EC is a heterogeneous disease with multiple histological subtypes and different treatment options. In establishing a diagnosis, clinical evaluation and imaging techniques (such as ultrasound and magnetic resonance imaging) are used, but the gold standard is histopathological examination [3,6,7,8].

The traditional histologic classification was elaborated by Bokhman, who divides EC into type I estrogen-dependent carcinomas (EEC): endometrioid adenocarcinoma and mucinous adenocarcinoma, and type II non-estrogen endometrial carcinomas (NEEC), which are less frequent but more aggressive subtypes, such as clear cell carcinoma, serous carcinoma, and carcinosarcoma (or malignant mixed Müllerian tumor or MMMT) [3,9]. This classification is important in making treatment decisions and establishing a realistic prognosis [3].

As the incidence of EC has increased, especially in postmenopausal women, the description and use of new, accurate prognostic tools and targeted therapies have been necessary. While early-stage EC generally has a favorable prognosis, high-grade, recurrent, or metastatic forms remain therapeutic challenges. New molecular analysis has identified four subtypes of endometrial cancer with an important role in prognosis, risk classification, and management, including target therapies in advanced/metastatic stages, but also in early stages [2,10]. These four molecular subtypes are POLE-mutated, mismatch repair-deficient (MMR-D), p53-mutated (p53abn), and no specific molecular profile (NSMP), based on molecular and genetic features. POLE-mutated and MMR-D generally have a good prognosis, while p53-mutated have a poorer prognosis compared to other subtypes. NSMP falls between the POLE-mutated and p53-mutated groups. This molecular classification can improve endometrial cancer management, leading to personalized and precision-based approaches [11,12].

The cancer stem cell (CSC) hypothesis has been proposed, which posits that a subpopulation of cells within a tumor possesses stem-like properties that contribute disproportionately to tumor initiation, progression, therapy resistance, and relapse. Thus, the ability to identify and characterize CSCs in EC offers new insights that are useful in predicting prognosis and choosing a targeted treatment strategy. While type I endometrial cancer is well-differentiated and has a better prognosis, type II is more aggressive, less differentiated, has less sensitivity to progesterone, and is often associated with a poor prognosis. Type II endometrial cancer is characterized by a higher proportion of cancer stem cells, which is why it is more likely to need stemness therapy [13]. This review summarizes the current knowledge regarding the identification, biological significance, prognostic impact, and therapeutic targeting of CSC populations in endometrial cancer.

## 2. Materials and Methods

**Figure 1:**
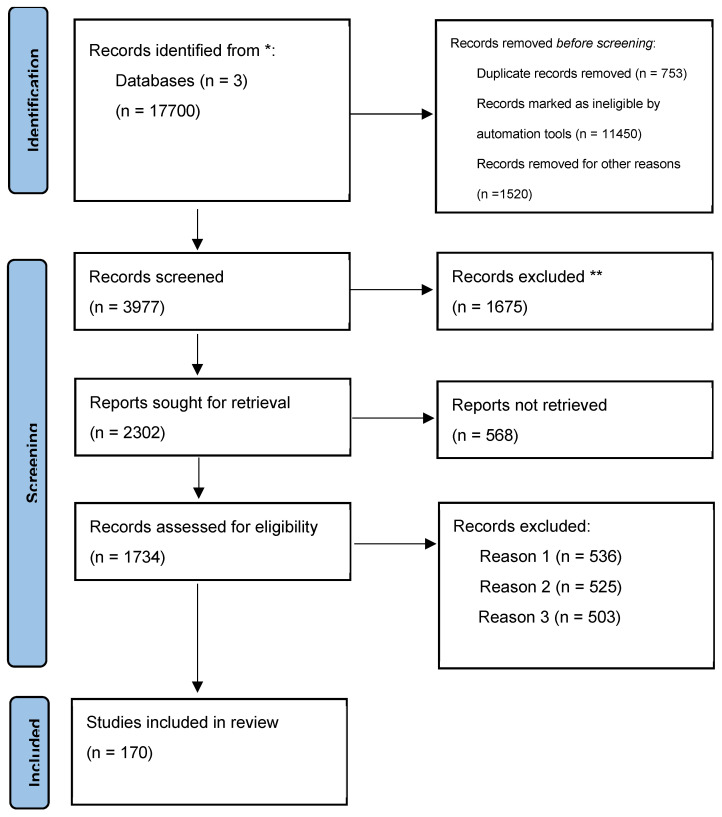
Flow diagram used for identification of studies in databases. * The number of records identified from Google Scholar, PubMed, and Cochrane databases. ** Records excluded by a human. Reason 1 Records excluded publications in a language other than English. Reason 2 Records excluded by a human reviewer due to inaccurate or inappropriate titles. Reason 3 Records excluded based on the study’s research design.

Due to the heterogeneity of the studies, a narrative review was conducted using Google Scholar, PubMed, and Cochrane databases to select relevant articles based on specific keywords: endometrial cancer; stemness; prognostic factors; targeted treatment; stemness markers; stemness pathway. We included manuscripts based on researchers’ efforts in identifying current knowledge about the CSC populations in endometrial cancer, their biological significance, prognostic impact, and therapeutic targeting, and 17,700 titles were obtained. Only manuscripts written in the English language were evaluated. Studies written in a language other than English were excluded. Also, duplicates were excluded after the initial search or in cases of missing data. Two authors, using PRISMA guidelines [14], independently screened relevant articles. We identified 162 potentially relevant articles. The limitation of our study is the different outcomes sought by the researchers and the heterogeneity of the methods used, which leads to limitations of general recommendations. The method used for selecting articles is presented in the following figure (Figure 1).

## 3. Defining Cancer Stem Cells in Endometrial Cancer

The biggest challenge in cancer therapy is tumor resistance to the classical approach. Thus, researchers’ interest has shifted toward the cancer stem cell population (CSC). CSCs are a small subpopulation of cancer cells within tumors with self-renewal, differentiation, and metastasis/malignant potential. They are involved in tumor initiation and development, metastasis, and recurrence. They were discovered in 1877 by Virchow’s student, Cohnheim, who pointed out that they possess an embryonic character [15,16]. Cell proliferation, invasion, metastasis, and angiogenesis generated by stem cells signaling pathways are activated in a similar way in cancer and during embryonic development. CSCs originate from adult tissue stem cells or differentiated cells [17].

These cells are believed to arise either from normal stem cells that acquire mutations or from differentiated cells that reacquire stem-like traits. Therapy against the tumor population alone has no or negligible effect on CSCs. CSCs, due to their stemness and therapeutic resistance, cause tumor relapse. Several predictive biomarkers to characterize CSCs have been sought, with important roles in diagnosis, prognosis, and as targets for therapy in patients [15,18]. These markers are not expressed in normal somatic cells and are thought to contribute to the stemness phenotype [16]. Stemness-related markers have been identified in embryonic stem cells (ESCs) and in adult stem cells, the two main types of human stem cells [19]. However, changes induced by radiotherapy, chemotherapy, or apoptotic tumor cells, which can alter the tumor microenvironment (TME), can induce CSCs with a high degree of plasticity, with changes in their phenotype and functional properties [15].

## 4. Identification and Characterization of CSCs in EC

Several markers and assays have been employed to isolate and study CSCs in EC:

Surface markers:
  CD133 (Prominin-1): Associated with high-grade tumors and a poor prognosis.
  ALDH1 (Aldehyde Dehydrogenase 1): Enzyme involved in detoxification; high activity correlates with stem-like phenotypes.
  CD44, CD117 (c-Kit): Involved in adhesion and signal transduction; implicated in EMT and metastasis.
Oncogenes and transcription factors:
  SOX2, OCT4, and NANOG: Pluripotency-related genes often upregulated in CSCs.
  Functional assays, pathways.
  Sphere formation assays.
  NF-kB, Wingless-INT (Wnt)/β-catenin, Notch1, and the HedghogPI3K-Akt-mTOR pathway.
  Aldefluor assay for ALDH activity.
  Side population analysis using Hoechst dye exclusion.

These tools enable the enrichment and functional validation of CSCs, though heterogeneity and overlap in marker expression remain challenges.

CSCs represent only a small fraction of heterogeneous cell populations within solid tumors (<1%), which makes their isolation a challenge [20]. Even so, CSCs have been isolated from various types of cancers [21,22,23]. Oncogenic somatic mutations likely have an important role in their formation [23,24,25]. Particular markers have been validated to differentiate CSCs in human cancers, including cell surface markers (CD9, CD24, CD133, CD44, EpCAM, etc.) and high enzymatic activities (aldehyde dehydrogenases—ALDH1) [23], as well as different oncogenes and transcription factors (transcription factors like Octamer binding transcription factor 4 (OCT), B Lymphoma Mo-MLV Insertion Region 1 Homolog (BMI1), North American Network Operations Group Homebox protein (NANOG), andsex-related high mobility group box (SOX)2, and SRY-Box 2 (SOX2)) [26]. Different stemness pathways (NF-kB, Wingless-INT (Wnt)/β-catenin, Notch1, HedgehogPI3K-Akt-mTOR, etc.) are involved in the stemness process. However, these markers are not specific to the CSC population of cancer cells [27]. This concept of cancer stem cells (CSCs) seems to be the most promising concept until now in understanding cancer resistance and progression [1,28]. In EC, CSCs contribute to cellular heterogeneity and exhibit increased resistance to standard treatments such as chemotherapy and radiation. Algorithms to construct a stemness subtype predictor will provide guidance for clinical management [29]. Therefore, targeting CSCs represents a new approach in the treatment of endometrial cancer in order to cure this disease [28].

### 4.1. Stemness-Related Surface Markers in Cancers

The identification of CSCs has relied on surface markers such as CD133, CD44, and the aldehyde dehydrogenase (ALDH) enzyme [29]. CD44 is a receptor for hyaluronic acid (HA), promotes chemo-resistance, and has anti-apoptotic properties on CSCs [28]. CD44 is a transmembrane glycoprotein with different roles in cell proliferation, migration, and adhesion. It mediates cell-to-cell communication [19]. CD133, another transmembrane glycoprotein and well-known biomarker, is associated with tumorigenicity, cell growth and development, and EMT [19,30,31,32,33]. There is evidence of co-expression of CD44 and CD133 in endometrial CSCs [2,34,35]. Multiple levels of heterogeneity characterize the tumor tissue, which contains CSCs [25]. CD133/CD44^+^ endometrial cancer cells were able to form tumor spheres, with high chemoresistance. Further, CD133/CD44^+^ endometrial cancer cells expressed the pluripotency markers Sox-2, Nanog, and Oct4 or other stemness-related genes with intense clonogenic ability [2].

Also, chemokines such as CXCL12 (SDF-1) and its receptor CXCR4 may regulate cell adhesion and gene transcription through different pathways [19].

It has been identified that ABCG2 proteins, a family of ATP transporters, facilitate the efflux of proteins, lipids, ions, and above all, an anti-cancer drug, inducing chemoresistance [2,36,37]. Aldehyde dehydrogenase 1 (ALDH1) is one of the enzymes involved in the oxidation of aldehyde [38], which is involved in stem cell differentiation [39]. It is correlated with a low prognosis in EC patients [2,40]. Its association with the tumor microenvironment (TME), stemness score, immune infiltration, and immunotherapy resistance has been explored. It can serve as a potential immunotherapy marker [41].

Programmed-cell death PD-1 and its ligand, PD-L1, have an intrinsic role as suppressors in aggressive EC cells [2]. Prominin 1 (*PROM1*) is considered a biomarker for cancer stem cells, with an unclear biological role, negatively associated with prognosis [42]. Bone morphogenic protein (BMP) triggers epithelial–mesenchymal transition (EMT) and enhances endometrial cancer cells’ proliferation [43]. Steroid receptor-associated and regulated protein (SRARP) exerts a tumor-suppressive effect by inhibiting the epithelial–mesenchymal transition via Wnt signaling in EC [44]. IRAK1 is a kinase involved in inflammation and innate immune response, as well as in cancer progression [45]. Focal adhesion kinase (FAK) is a tyrosine kinase that regulates cell adhesion and migration [46]. TRIM28 (tripartite motif protein 28) plays an important role in deoxyribonucleic acid (DNA) damage repair and the maintenance of cancer cellular stemness [47]. Leukemia inhibitory factor receptor (LIFR) plays an important role in cancer development, metastasis, and therapy resistance, but little is known about its biological significance, even though it was demonstrated to be associated with poor progression-free survival [48].

CSCs have the ability to form spheroid cells. Understanding of spheroid cells may help to find novel CSC markers [49].

### 4.2. Stemness-Related Pathways

The proper functioning of normal stem cells (SCs) is absolutely essential for embryonic development and tissue homeostasis throughout life [15,50]. The replicative and regenerative potentials of SCs have been postulated, wherein cancer may also develop from a subset of cells with SC-like properties [2]. These cells are required for tumorigenesis [21,27]. In addition to their cancer-initiating ability, CSCs play a critical role in modulating other processes, such as EMT [29] and drug resistance [28].

Pathways frequently identified in cancers display aberrant activities in cells selected for stemness [51]. There is current evidence for Notch, Hedgehog (Hh), PI3K/mTOR, and Wnt/β-catenin pathway regulation in CSCs [19].

#### 4.2.1. (Hh) Pathway

The Hedgehog (Hh) pathway is an important regulator in embryonic development, with important roles in cell proliferation, cell differentiation, tissue polarity, and stemness maintenance [19,52]. The Sonic Hh Hedgehog pathway is involved in tissue repair and the regeneration of epithelial cells [53,54,55]. There is evidence that this pathway (Hh) is affected in various cancer types [56]. And its activation plays an important role in the epithelial-to-mesenchymal transition and cell adhesion [53,57].

#### 4.2.2. Notch Pathway

Notch signaling plays a critical role in stemness and angiogenesis. It is involved in cell-to-cell communication and homeostatic processes [19,58,59]. Activating this signaling pathway can have very diverse effects, depending on the signal dose. Its deregulation can be created by mutations, overexpression, inactivation, or epigenetic alterations functioning as an oncogene [53].

#### 4.2.3. PI3K/Akt/mTOR Pathway

The phosphatidylinositol-3-kinase (PI3K)/Akt and the mammalian target of rapamycin (mTOR) signaling pathways are crucial for stemness. In human cancers, its deregulation is involved in stem cell proliferation and differentiation [19,60]. Phosphatase and tensin homolog deleted on chromosome 10 (PTEN) is a tumor suppressor known for its negative regulation of PI3K/AKT cascades [31,61,62].

#### 4.2.4. Wnt/β-Catenin Pathway

Wnt pathways also play key roles in stem cell pluripotency [19,63], DNA hypomethylation, and WNT-Integrin [64]. Frizzled 2 (*FZD2*) is an important receptor in the Wnt pathway, with high expression in malignant tumors, and it is related to prognosis. Expression was related to patient overall survival (OS), disease-free survival, tumor microenvironment, immune cell infiltration, and tumor cell stemness [58,60,65,66,67]. There are currently compounds in development to specifically target the Wnt pathway [18,58].

#### 4.2.5. NF-κB in Endometrial Cancer Stem Cells

The nuclear factor "kappa light chain enhancer" of activated B-cells (NF-κB) plays a critical role in the progression of cancer [68]. It has been studied for its involvement in inflammation; this signaling pathway also has a role in cancer development. Proinflammatory cytokines activate the NF-κB signaling pathway involved in cancer progression [53]. Different cellular signaling pathways are known to modulate the response to radiotherapy in endometrial cancer, including the PI3K/AKT and NF-κB pathways, in association with DNA damage repair mechanisms and the immune system [69].

#### 4.2.6. Epithelial–Mesenchymal Transition (EMT)

(EMT) promotes the acquisition of mesenchymal features of epithelial tumor cells; reduces cell-to-cell adhesion and cell polarity; reduces the expression of epithelial markers, for example, E-cadherin; and enhances mesenchymal markers, including N-cadherin. EMT stimulates cell migration, contributing to invasion, metastasis, and resistance to chemotherapy and radiation [2,70]. We found that EMT is consistent with the activity of several developmental pathways, including the Notch and Wnt pathways, significantly correlated with poor survival [71].

#### 4.2.7. Hypoxia and Oxidative Stress

In particular, the increased lipogenic activity and the alteration of metabolic homeostasis due to interactions between p53 and AMP-activated protein kinase (AMPK), NF-kB, and HIF-1α favor metabolic reprogramming and the Warburg effect observed in cancer cells as a consequence [72]. The HIF-1 pathway is activated in endometrial cancer stem cells [49].

A high concentration of reactive oxygen species (ROS) is detrimental to the cell. ROS promotes DNA alteration and protein and lipid modifications. It is already known that cancer cells, because of altered oncogenic signaling pathways and active metabolism, are characterized by a high ROS level. Cancer stem cells are able to adapt [73,74]. Oxidative stress is involved in cell proliferation, invasion, metastasis, apoptosis evasion, and angiogenesis. EMT could be regulated by ROS through activation of the NF-kB signaling pathway, involved in metastasis through the Wnt signaling pathway [53,75,76]. Cancer cells are adapting to oxidative stress through the Warburg effect, switching from aerobic glycolysis to anaerobic glycolysis. This process is independent of oxygen available in the microenvironment and leads to low ROS level formation [18,53,77,78].

#### 4.2.8. The Tumor Microenvironment (TME)

The multilineage differentiation capacity of CSCs may allow them to take part in tumor angiogenesis in the TME. Recent research has shed light on the relationship between CSCs and the vascular microenvironment [64]. Stemness is enhanced by immune suppression in the tumor microenvironment (TME), hypoxia, and extracellular cell matrix (ECM) degradation [62,79].

Multifactorial functions of TGFβ signaling in cancer, including regulation of the tumor microenvironment (TME) and the behavior of cancer cells, play a key role in cancer stemness. TGFβ-inhibition strategies to restore the extracellular matrix (ECM) can reverse epithelial–mesenchymal transition (EMT) and enhance cancer chemotherapy efficacy [80].

#### 4.2.9. Autophagy

Autophagy is a mechanism activated in stress conditions, such as hypoxia, that maintains cell survival. Treatment resistance is activated through autophagy in the case of conventional anticancer therapies. PI3K/AKT/mTOR and p53 signaling pathways can inhibit the process of autophagy [81]. The status of p53 currently constitutes one of the most relevant criteria to understand the role of autophagy as a survival mechanism in cancer [72].

### 4.3. Stemness-Related Oncogenes

Stemness-related genes—notably SOX2 (sex-determining region Y-box 2), OCT4, and NANOG—are responsible for maintaining the stem-cell-like phenotype, including conserving self-renewal capacity and pluripotency [82].

Frequent mutations occur in the genome, and cancer-related oncogenes are involved in this process. Blocking the activity of oncogenes can be useful as a specific targeted treatment for tumor cells. Oncogenes and their target cells interact, inducing reprogramming of the epigenome through stem cell reprogramming. SOX2 is a multifunctional proto-oncogene that is closely associated with stemness and EMT, while OCT4 is important in the maintenance and restoration of CSC pluripotency [83]. SRY-box transcription factor 11 (*SOX11)* is a transcription factor that has recently been found to be a prognostic marker for certain cancers. It is correlated with survival outcomes, clinical features, and stemness [84].

Moreover, the upregulation of Nanog, Oct4, and Sox2 in CSCs inhibits apoptosis through various signaling pathways, such as the Sox2/ORAIL/STIM1 and Oct4/Tcl1/Akt1 pathways [83,85,86]. An analysis of patient data suggested a stronger role for SOX2, relative to OCT4 or NANOG, for tumor relapse potential. SOX2 may be a consistent indicator of tumor repopulation following chemotherapy [30].

Potassium Inwardly Rectifying Channel Subfamily J Member 14 (KCNJ14) is one of the cancer genome’s least investigated genes that is involved in cell survival, RNA modification, and cancer stemness [87]. Also, the loss of the tumor suppressor, PTEN (phosphatase and tensin homolog), is well studied in endometrial cancer [88]. It is a tumor suppressor gene that regulates several crucial cell functions, such as proliferation, survival, and migration through phosphatidylinositol 3-kinase (PI3K)-dependent and -independent mechanisms. PTEN remains a controversial prognostic/predictive biomarker [89]. EC may be caused by mutations in PIK3CA, PTEN, CTNNBI.3, ARID1A, and K-RAS genes [15,90].

Nuclear factor erythroid 2 (NFE2)-related factor 2 (NFE2L2, or NRF2) is a transcription factor that affects the expression of antioxidant genes, with a significant role in the redox balance, crucial in cancer cells [91].

KRAS mutation plays an important role in EC, with prognostic influence, helping to find therapeutic personalized options to treat cancer [92]. γ-glutamyl hydrolase (GGH) affected differentiation, cancer proliferation, and immune regulation. GGH may influence the progression of EC by regulating the glycolytic process [93].

When identifying messenger RNA (mRNA), the expression-based stemness index of (mRNAsi-)-related genes of endometrial cancer samples was significantly higher than that of normal samples and was related to the International Federation of Gynecology and Obstetrics (FIGO) stage, pathological grade, postoperative tumor status, and overall survival of endometrial cancer patients. Endometrial cancer cell stemness was related to patient prognosis [22].

Gene public databases can be used to measure the gene expression profiles of tumors [23].

## 5. Prognostic Implications of CSCs in EC

Numerous studies have demonstrated that increased expression of CSC markers correlates with adverse clinicopathological features:

Advanced FIGO stage.
Deep myometrial invasion.
Lympho-vascular space invasion.
Higher recurrence and metastasis rates.
Poorer overall survival (OS) and disease-free survival (DFS).

For example, high ALDH1 expression is an independent predictor of reduced DFS and OS. CD133+ cell populations have been linked to therapy resistance and increased tumor-initiating potential in xenograft models.

The purpose of this study was to investigate the correlation between stemness markers (CD44 and CD133) and prognostic value in endometrial cancer (EC). High expression of CD44 and CD133 was closely related to the adverse prognosis of early-stage EC patients [94]. These findings indicate the crucial roles of CD44 and CD133 in EC pathogenesis, highlighting their potential as biomarkers and therapeutic targets in this malignancy [95].

Regulatory networks that control cellular plasticity can re-emerge after tissue injury. One such regulatory molecule is the cell surface ectoenzyme ecto-5′-nucleotidase, which is associated with worse clinical outcomes [19].

ALDH cells demonstrated greater endometrial cancer stem cell activity than CD133^+^ cells and had increased expression of stem cell and epithelial–mesenchymal transition genes, showing higher prognostic potential [63]. High ALDH1A1 expression was associated with poor survival. Similarly to ALDH inhibition, GLUT1 inhibition synergized with paclitaxel to block endometrial cancer proliferation [25].

Recent discoveries indicate a distinct role for PD-L1 in modulating epithelial-to-mesenchymal transition (EMT), the cancer stem cell (CSC)-like phenotype, metastasis, and resistance to therapy [79,96]. Activation of OCT4 signaling and upregulation of EMT induce PD-L1 expression in cancer cells, and a possible immune evasion mechanism seems to be employed by cancer stem cells during metastasis [70]. The high expression level of Coatomer protein complex Zeta 1 (COPZ1) in tumors, COPZ1, was associated with poor survival, indicating an important clinical prognostic value [79].

Downregulation of EpCAM, a transmembrane glycoprotein, favors a poor prognosis and cancer cell invasion of EC. However, its correlation with EC is not clear. These findings provide new insights into the prognostic role of EpCAM in patients with EC [97].

Transcriptional targets linked to chemoresistance in tumors can be found using bioinformatics analysis [68]. Diverse gene mutations, such as in KRAS, PTEN, and PIK, may lead to EC through PI3K/Akt/mTOR pathway activation [31]. This process is related to prognosis. Also, a connection between chemoresistance and the CSC phenotype can be demonstrated with these gene mutations [68].

The WNT pathway in tumorigenic tissue compared with non-tumorigenic tissue is a valuable biomarker for risk stratification and predicts the prognosis of patients with cancer [98]. It is also intensely correlated with expression of E-cadherin and β-catenin [99]. Immunotherapy through monoclonal antibodies has been developed, such as WNT signaling pathway inhibitor 1 (DKK1) and DKN-01. This can be used as a predictive biomarker for evaluating pembrolizumab treatment [53,60,100].

Nuclear factor kappa B (NF-κB), a transcription factor, is associated with prognosis in a variety of human cancers and is a potentially unfavorable prognostic marker [101]. It has been also investigated for its role in predicting the efficacy of some therapies in advanced colorectal cancer [53,102,103].

Regarding the Notch signaling pathway’s prognostic value, its high mRNA expression can be used as bad overall survival (OS) prognostic factor in cancers, with high accuracy [53,104,105,106].

The prognostic significance of Hh protein overexpression was not consistent among different studies [107], while, according to other studies, increased Hh activity correlates with tumor immunosuppression across diverse cancers, being not only a predictive biomarker for chemotherapy resistance but also better able to predict clinical outcomes in combination with PD-L1 expression [108]. The majority of genes participating in autophagy, such as Hh, have a prognostic value [53,109].

Related to the PI3K/mTOR signaling pathway and its value as a prognostic factor, positive expression of the AKT, mTOR, and P70S6K proteins was found in patients with advanced-stage cancer, with low differentiation and metastasis [110]. Ubiquitin-conjugating enzyme E2S (UBE2S), a potential oncogene related to the PI3K/AKT/mTOR pathway, could be involved in apoptosis, proliferation, and migration, and it is valuable as a biomarker in the prognosis of cancer [31,111,112,113]. PRMT6 is associated with EC via AKT/mTOR signaling and has major prognostic value [114].

### Stemness-Related Genes as Prognostic Factors

The Cancer Genome Atlas database was used for the evaluation of 13 CSC markers (ALDH1A1, CD44, EPCAM, KIT, LGR5, NES, NOTCH3, POU5F1, PROM1, PTTG1, ROR1, SOX9, and THY1). Models used to predict chemotherapy response and survival were built. ALDH1A1 and LGR5 mRNA expressions indicated a higher platinum sensitivity [115]. It was demonstrated that CTNNB1 is a key factor in the interaction of the canonical Wnt signaling pathway with 10 upregulated cancer-associated genes, which reveals efficient biomarkers that can be used for the prognosis of cancers [53,116].

Overexpression of Sex-determining region Y-box 2 (SOX2) is significantly correlated with advanced histological grade and poor prognosis [88].

SOX2 overexpression significantly correlated with poor prognosis, and targeting the biological pathways of SOX2 seems to be a promising option in developing relevant therapeutic interventions [117].

While some studies observed that high SRY sex--related high mobility group box (SOX)2, SOX2 expression was significantly associated with poor overall survival [118], according to other studies, the prognostic value of CD133 and SOX2 expression in advanced cancer remains unclear [119].

Researchers observed that lncRNAs/SOX2 axes hold immense potential and could ultimately be used to improve the survival and prognosis of cancer patients [120].

The stem signatures associated with antibodies of TP53 and SOX2 help to predict survival and prognosis in solid cancers. The identification of potential immunotherapeutic targets will yield early and effective treatments [121].

OCT4 is one of the most important regulators in pluripotency and correlates with worse cancer outcome in most tumors and poor disease-free survival, having a prognostic value [122,123].

In the same way, a combination of NANOG and OCT4 may be a predictor for cancer recurrence [124,125]. Homeobox protein NANOG (hNANOG), a known stem cell marker, is a downstream effector of Gli; the expression of Gli and NANOG predicted poor patient prognosis [126,127].

WDHD1 mRNA levels were significantly increased in many cancers. WDHD1 expression’s prognostic value lies in its association with significantly shorter overall survival (OS) in patients [128].

Potassium Inwardly Rectifying Channel Subfamily J Member 14 (KCNJ14) is one of the cancer genome’s least investigated genes, but using several databases, it has been found to be involved in cancer stemness. Based on these novel findings, KCNJ14 may be a useful independent prognostic biomarker for a range of cancers [87].

Ephrin receptor B2 (EPHB2) seems to be linked to genome instability, but its importance in cancer progression remains unclear. Recent studies indicated that EPHB2 is involved in cellular proliferation, invasion, and resistance to anti-cancer immune responses, as well as M2 macrophage infiltration [129].

Another potential systemic approach is to investigate estrogen-response-related genes in EC and therapeutic responses [130].

Gene Set Enrichment Analysis showed that the BTLA B and T-lymphocyte attenuator (BTLA) and its co-expressed genes mainly act through pathways, including immune response regulation, cell surface receptor signaling pathway, antigen receptor-mediated signaling pathway, or antigen binding and leukocyte migration. BTLA has potential as a prognostic marker for many cancers. BTLA has a close relationship with the development of tumors, and targeting BTLA is worthy of further analysis [131]. These markers and their prognostic roles are presented in a table below (Table 1).

## 6. Therapeutic Targeting of CSCs in Endometrial Cancer

Conventional therapies often fail to eliminate CSCs, leading to disease relapse. Targeted strategies are under development:

a. Signaling pathway inhibition:
  Wnt/β-catenin, Notch, and Hedgehog pathways are critical in maintaining CSC stemness.
  Inhibitors targeting these pathways (e.g., Notch inhibitors) show promise in preclinical models.
  PI3K/AKT/mTOR pathway modulation: Frequently dysregulated in EC; dual inhibition may suppress both CSC and non-CSC populations.
b. Epigenetic therapies:
  Downregulate stemness-related gene expression.
c. Immunotherapeutic strategies targeting stemness surface markers:
  Emerging interest in CAR-T cell therapy targeting CD133+ CSCs.
  Immune checkpoint inhibitors may modulate the tumor microenvironment to reduce CSC survival.
d. Combination therapies:
  CSC-targeted agents combined with standard chemotherapy/radiotherapy show synergy in eliminating tumor bulk and CSCs.

### 6.1. Targeting of Stemness Pathways

The combined inhibition of different pathways with platinum treatment is suggested as a new treatment for cancer, increasing the sensitivity of CSC [68].

Various methods of targeting Wnt pathways are being sought by researchers. The inhibition of porcupine required for Wnt activation, induced by LGK974, was shown in preclinical models. A monoclonal antibody against Wnt signaling pathway inhibitor 1 can be used as a therapy in patients with endometrial cancer [53,60]. Wang et al., in a study on 21 patients, showed that a synthetic form of progesterone has a beneficial effect in early endometrial carcinogenesis by inhibiting Wnt/β-catenin signaling gene expression. IUD levonorgestrel is one of the most efficient forms of progesterone therapy [135]. Wnt-driven cancers can be targeted at many points in the pathway [60,136]. Niclosamide (trade name Niclocide), an FDA-approved salicylamide derivative used for the treatment of tapeworm infections, can be used to target the Wnt/β-catenin pathway [18,60,137,138]. Salinomycin, an antibiotic potassium ionophore, is a selective inhibitor of cancer stem cells [137]. It also induces apoptosis; interferes with Wnt/β-catenin signaling; inhibits proliferation, invasion, and migration [49,60,137]. It is combined with the FDA-approved antiparasitic drug Ivermectin [139,140,141], which suppresses tumor metastasis through inhibition of the WNT/β-catenin pathway [80]. Mebendazole and Albendazole, other anthelmintic drugs, have also been investigated for the inhibitory effects of WNT signaling [137]. The steroidal anti-inflammatory drug Tolfenamic Acid was found to inhibit cancer cell proliferation by acting on the WNT pathway [142]. Quercetin (polyphenole) and Resveratrol are polyphenolic compounds that modulate long non-coding metastasis through Wnt signaling pathways [137].

Nuclear factor kappa B (NF-κB), *NF-kB1,* plays an important role in the immune responses, but it is also involved in the processes of oncogenesis and DNA repair, also acting as a predictive and prognostic marker in the treatment of cancer [143]. However, NF-κB inhibitors are useful in association with other chemotherapies [144]. Thalidomide acts in this way [145,146]. Bortezomib has remarkable antitumor activity in different cancers [50].

*Notch3’s* mRNA high expression was demonstrated to have prognostic value for better OS for cancer patients [53,147].

To target the Notch signaling pathway, two groups of inhibitors were used: monoclonal antibodies against Notch ligand receptor (DLL-4) (mAbs) and γ-secretase inhibitors (GSIs). Enoticumab is a mAb against DLL-4 that showed efficacy in advanced cancers. Demcizumab is a DLL-4 antibody that showed low efficacy in cancer treatment, and it is not used in clinical practice [53,104,105]. All these inhibitors inhibit cancer proliferation, angiogenesis, metastasis, and stem cell marker expression through Notch signaling pathway inhibition [53,104,105]. Non-coding RNAs, antibodies, and antibody–drug conjugates, as treatment strategies to block Notch3 signaling, can provide precise targeted cancer therapy [58].

Hh pathway inhibitors can ameliorate tumors, but their efficacy depends on the alterations encountered in the signaling pathway [148]. Among all proteins involved in the signaling cascade, smoothened (SMO) and glioma-associated oncogene homolog (GLI) Gli transcription factors are the main targets studied by researchers. Vismodegib, a cyclopamine derivative, showed efficacy in metastatic cancer treatment. Sonidegib is another SMO antagonist used in advanced cancer. The GLI inhibitor Genistein (phase I and II), an isoflavone, has been shown to be effective in treating different tumors [149,150].

SHh Inhibitors: Inhibitors of Sonic Hedgehog (SHh) were used in preclinical trials in mice, with good results [53,149].

New clinical trials with smoothened (SMO) antagonists did not show much success in cancers. These studies suggest that the Hh pathway involves multiple mechanisms of activation or inhibition, which makes this pathway extremely complex. The SMO-specific antagonists may not stop all relevant pathways, and resistance may develop. A multitarget profile or a combination of drugs could be a promising solution for achieving clinical response [52].

The hypoxia in the TME helps maintain the stemness of cancer stem cells (CSCs), promoting the invasion and metastasis of cancer cells. Drugs that target CSCs, such as metformin, may exhibit efficacy similar to anti-angiogenic therapies, which is due to the reduction in the proportion of CD44^+^/CD117^+^ CSCs and the alleviation of hypoxia in the tumor microenvironment [133,151]. Using ROS scavengers is another strategy to decrease ROS in cancer cells [53].

Bimiralisib (PQR309), an inhibitor of PI3K and mTOR, has been studied in clinical trials [112]. In a phase II clinical trial, mTOR inhibition showed a significant effect on EC treatment [31,113,152]. Metformin is a drug that inhibits the mTOR pathway by activating adenosine monophosphate-activated protein kinase (AMPK) [31,153]. Everolimus (RAD001) was found to induce apoptosis in cancers by upregulating apoptotic gene expression [154,155]. Ropivacaine restrained cancer cell stemness by inactivating the PI3K/AKT signaling pathway [156].

PRMT6 exerts oncogenic effects via activation of the AKT/mTOR pathway. It can be targeted by miR-372-3p in EC. Inhibition of AKT/mTOR signaling by rapamycin attenuates PRMT6-mediated EC progression [114]. Finally, dietary compounds and microRNAs seem to have an important role in autophagy modulation. But the data about autophagy is lacking in endometrial cancer [81]. A PARP inhibitor, Olaparib, combined with the AKT inhibitor capivasertib, assesses molecular markers of response and resistance. Tumor samples acquired pre- and on-therapy can help predict patient benefit [157].

TGFβ inhibitors are currently in phase II and phase III clinical trials, with both positive and negative clinical outcomes having been observed [80]. Regarding the mechanism of action, metformin treatment remarkably downregulated IL-6/STAT3 signaling activity, which subsequently resulted in VEGF and TGFβ1 expression [158].

Furthermore, endometrial cancer stem cells show metabolic plasticity. This characteristic is crucial to their resistance. Immune checkpoint inhibition is another clinically relevant targeting strategy, such as targeting PD-L1 in EC with promising anti-tumor effects [28].

Pacritinib is an IRAK1 inhibitor used to upregulate PD-L1 expression and inhibit the proliferation of cancer cell lines. Moreover, low IRAK1 expression can function as a prognostic marker in different cancers [45]. Another strategy is using the LIFR inhibitor EC359, which diminishes the stemness of cancer cells [48].

### 6.2. Targeting of Stemness Genes

Up-frameshift mutant 1 (UPF1) is a protein associated with the mRNA degradation pathway, which is significantly associated with cancer stemness. UPF1 also acts as an RNA-binding protein (RBP), playing an important role in gene regulation. Long noncoding RNAs (lncRNAs) modulate CSC characteristics. A newly identified lncRNA, LINC00963, was reported as upregulated in CSCs. The inhibition of UPF1 and LINC00963 severely affected the tumorigenic potential of ECSCs. The authors have demonstrated that the UPF1/LINC00963/miR-508-5p/SOX2 axis has potential value in modulating chemoresistance and tumor progression in EC [159,160,161].

Overexpression of SOX2 is significantly correlated with advanced histological grade, poor prognosis in EC, and aggressive behaviors in ECSCs [162]. StarBase analysis showed that miR-508-5p had binding sites with SOX2, which can be further explored by targeting SOX2 in ECSCs [159].

Also, treatment with EC359 reduced the spheroid formation of EC cancer stem cells and reduced the levels of cancer stem cell markers SOX2, OCT4, and NANOG [163].

Leukemia inhibitory factor (LIF) and its receptor (LIFR) play a major role in cancer stemness, progression, metastasis, and therapy resistance. A novel LIFR inhibitor, EC359, resulted in the induction of apoptosis and reduced the levels of cancer stem cell markers SOX2, OCT4, and NANOG in EC [163].

OCT4 is one of the most important regulators in pluripotency, and targeting OCT4 could be a potential therapeutic management in cancers. Studies have reported that CCL16 protein decreases OCT4 expression and reduces the ALDH+ subpopulation [164].

Nanog expression appears to be related to poor prognosis in cancers, and iron chelators may offer a novel therapeutic strategy [165]. Targeting Gli may reduce the stemness of cancer cells via indirect targeting of NANOG [126]. The stemness of CSCs is maintained by upregulating NANOG, which subsequently activates the JAK/STAT3 pathway, supporting its potential as a prognostic biomarker and therapeutic target in this disease [127].

WDHD1 mRNA participates in the processes of DNA replication and DNA damage repair. It has also been found that WDHD1 is involved in tumor stemness, RNA methylation modification, and tumor progression. This theory provides theoretical support for future WDHD1-targeted therapies [128]. As a consequence of miRNAs’ functional roles in cancer cells and absence in normal tissue, they can be used as a potential novel therapeutic target for the high-risk endometrial cancer patient population [134]. Therapeutic options are presented in the table below (Table 2).

Some new drugs are used in different clinical trials and are examples of future directions. OMP-54F28 was studied as a therapeutic option in phase I clinical trials for different types of cancer [166]. LGK974 is a porcupine inhibitor acting against the Wnt signaling pathway, which has been demonstrated to have antitumoral effects in phase I clinical trials [167]. Phase I studies have also been conducted with bardoxolone methyl (RTA-402) against Nf-kB for various types of cancer [168]. γ-Secretase Inhibitors (GSIs) are critical for Notch signaling pathway inhibition, such as MK-0752, which is in phase I clinical testing for breast cancer [169].

## 7. Limitations and Future Perspectives

While around 20% of type I EC can still recur and become resistant to treatment, type II is known for its higher rate of chemoresistance, up to 50%, with a higher proportion of stemness gene expression [170].

Despite the progress in understanding EC-CSCs, several challenges remain:
  A lack of universally accepted markers for CSC identification.
  Functional heterogeneity among CSC subpopulations.
  Insufficient clinical trials targeting CSCs specifically.
  Difficulty in tracking CSC dynamics in vivo.
Future directions include the following options:
  The development of liquid biopsy techniques for CSC detection.
  The integration of single-cell RNA sequencing to map CSC subclones.
  Clinical validation of CSC-targeted therapies in combination with conventional modalities.

## 8. Conclusions

Failure of conventional therapy in advanced or metastatic cancers represents an important burden in oncology. CSCs in endometrial cancer represent a pivotal component of disease aggressiveness and therapeutic resistance. Their identification and targeting hold the key to personalized, effective treatment regimens. Continued research into CSC biology, combined with translational and clinical studies, may revolutionize the management of aggressive and recurrent EC. This highlights their role in prognosis and targeted treatment as well.

But the plasticity of cancer stem cells still represents an escape mechanism leading to therapy resistance; unfortunately, its inhibition is likely to result in adverse events. A major future challenge is understanding the involvement of this issue in metastasis and therapy resistance.

## Figures and Tables

**Table 1:** Stemness markers and their prognostic role.

Articles	Markers	Prognosis
[68,94,132]	CD44, CD133	Adverse prognosis of early-stage EC
[131]	Cell surface ectoenzyme ecto-5′-nucleotidase	Worse clinical outcomes through cellular plasticity
[70,133]	ALDH1A1	Poor survival
[70]	PD-L1	Metastasis, immune evasion
[79]	COPZ1	Poor survival
[97]	Downregulation of EpCAM	Poor survival
[31,68]	Notch, PIK3CA, PIK3R1, KRAS, PTEN	Prognostic factors, poor survival
[53,60,100]	WNT pathway	Prognostic factor valuable in risk stratification
[53,60,100]	A monoclonal antibody against dickkopf WNT signaling pathway inhibitor 1 (DKK1) and DKN-01	Predictive biomarker in EC treatment
[102,103]	NF-κB	Predicting efficacy of some therapies
[53,104,105,106]	Notch	Worsen overall survival (OS)
[53,109]	Hh	Prognostic value in evaluating autophagy
[31,110,111,112,113,133,134]	PI3K/mTOR	Poor prognosis
[115]	LGR5 mRNA in relationship with ALDH1A1	Indicates higher sensitivity to platinum-based therapy
[53,116]	CTNNB1 in interaction with Wnt	Prognostic value
[118,119,120]	SOX 2	Advanced histological grade and poor prognosis
[121]	Stem signatures associated with antibodies of TP53 and SOX2	Prognostic value
[122,123]	OCT4	Poor disease-free survival
[124,125]	NANOG and OCT4	Predictor for cancer recurrence
[126,127]	NANOG as downstream effector of Gli	Poor prognosis
[128]	WDHD1 mRNA	Shorter overall survival (OS)
[87]	KCNJ14	Prognostic value
[129]	EPHB2	Prognostic value
[130]	Estrogen-response-related genes	Therapeutic responses
[131]	BTLA B and T-lymphocyte attenuator (BTLA)	Prognosis

COPZ1 = coatomer protein complex Zeta 1—all markers level is upregulated in poor prognosis, except EpCAM; NF-κB = nuclear factor kappa B; KCNJ14 = Potassium Inwardly Rectifying Channel Subfamily J Member 14; EPHB2 = Ephrin receptor B2; BTLA = BTLA B and T-lymphocyte attenuator, NANOG homebox protein; OCT4 = octamer-binding protein 4; SOX2 = sex-determining region Y-box 2; ALDH1A1 = aldehyde dehydrogenase 1A1; Pi3K/mTOR = phosphatidylinositol-3-kinase/mammalian target of rapamycin; Hh = Hedgehog; Wnt = wingless; PTEN = phosphatase and tensin homolog; PD-L1 = death protein ligand 1.

**Table 2:** Therapeutic stemness targeting.

Articles	Markers Targeted	Therapy
[53,60]	Wnt signaling pathway	A monoclonal antibody against dickkopf WNT signaling pathway inhibitor 1
[60,135,136]	Wnt signaling pathway	Progesterone
[18,60,137,138]	Wnt signaling pathway	Niclosamide
[49,60,137]	Wnt signaling pathway	Salinomycin
[80,140,141]	Wnt signaling pathway	Ivermectin
[137]	Wnt signaling pathway	Mebendazole and Albendazole
[137,142]	Wnt signaling pathway	Tolfenamic Acid
[137]	Wnt signaling pathway	Quercetin and Resveratrol
[53,143,145,146]	NF-κB signaling pathway	NF-κB inhibitors
[53,145,146]	NF-κB signaling pathway	Thalidomide
[53]	NF-κB signaling pathway	Bortezomib
[53,58,104,105,147]	Notch signaling pathway	Enoticumab, Demcizumab, or other Notch inhibitors
[52,53,148,149,150]	Sonic Hedgehog signaling pathway	Vismodegib, Sonidegib, Genistein
[151]	CD44^+^/CD117^+^ CSCs	Metformin
[53]	Hypoxia	ROS scavengers
[31,112,113,152]	PI3K/AKT signaling pathway	Bimiralisib
[31,153]	PI3K/AKT signaling pathway	Metformin
[154,155]	PI3K/AKT signaling pathway	Everolimus
[156]	PI3K/AKT signaling pathway	Ropivacaina
[151]	Autophagy	Dietary compounds
[157]	PI3K/AKT signaling pathway	Olaparib combined with capivasertib
[80,158]	TGFβ	TGFβ inhibitors
[28]	PD-L1	Inhibitors
[45]	PD-L1	Pacritinib
[163]	LIFR	EC359
[159,160,161]	Up-frameshift mutant 1 (UPF1)	UPF1inhibitor
[163]	SOX2, OCT4, NANOG, acting on LIF	EC359
[164]	OCT4	CCL16 protein
[165]	Nanog	Iron chelators
[126]	Nanog	Gli inhibitors

OCT4 = octamer-binding protein 4, SOX2 = sex-determining region Y-box 2, Pi3K/mTOR = phosphatidylinositol-3-kinase/mammalian target of rapamycin, Hh = Hedgehog, Wnt = wingless, NF-κB = nuclear factor kappa B, PD-L1 = death protein ligand 1, UPF1 = up-frameshift mutant 1, TGFβ transforming growth factor, LIF = leukemia inhibitory factor receptor.

## Data Availability

No new data were created or analyzed in this study. Data sharing is not applicable to this article.

## Abbreviations

The following abbreviations are used in this manuscript:

RT-PCR	Reverse transcription polymerase chain reaction.
CSC	Cancer stem cell population.
SOX2	Sex-determining region Y-box 2.
NANOG	Nanog homeobox.
OCT4	Octamer-binding protein 4.
EC	Endometrial cancer.
ECIS	European Cancer Information System.
IARC	International Agency for Research on Cancer.
MMMT	Malignant mixed Müllerian tumor.
ESCs	Embryonic stem cells.
TME	Tumor microenvironment.
ALDH 1	Aldehyde dehydrogenase.
HA	Hyaluronic acid.
ABC	ATP-binding cassette.
PD-1	Programmed-cell death.
PROM1	Prominin 1.
BMP	Bone morphogenic protein.
EMT	Epithelial–mesenchymal transition.
SRARP	Steroid receptor-associated and regulated protein.
FAK	Focal adhesion kinase.
DNA	Deoxyribonucleic acid.
LIFR	Leukemia inhibitory factor receptor.
PI3K/Akt	Phosphatidylinositol-3-kinase (PI3K)/Akt.
Hh	Hedgehog.
mTOR	Mammalian target of rapamycin.
PTEN	Phosphatase and tensin homolog.
OS	Overall survival.
TLRs	Toll-like receptors.
TNF-α	Tumor necrosis factor alpha.
TGF	Beta transforming growth factor.
AMPK-	Activated protein kinase.
ROS	Reactive oxygen species.
RNA	Ribonucleic acid.
ECM	Extracellular cell matrix.
EMT	Epithelial–mesenchymal transition (EMT).
SRY-	Box transcription factor 11 (*SOX11).*
KCNJ14	Potassium Inwardly Rectifying Channel Subfamily J Member 14.
NFE2L2, or NRF2	Nuclear factor erythroid 2 (NFE2)-related factor 2.
GGH	γ-glutamyl hydrolase.
mRNA	Messenger RNA.
FIGO	International Federation of Gynecology and Obstetrics.
CGA	The Cancer Genome Atlas.
Wnt	Wingless.
NF-κB	Nuclear factor kappa B.
GLI	Glioma-associated oncogene homolog.
SMO	Smoothened.
SHh	Sonic Hedgehog.
UBE2S	Ubiquitin-conjugating enzyme E2S.
AMPK	Adenosine monophosphate-activated protein kinase.
UPF1	Up-frameshift mutant 1.
RBP	RNA-binding protein.
lncRNAs	Long noncoding RNA.
COPZ1	Coatomer protein complex Zeta 1.
EPHB2	Ephrin receptor B2.
GSEA	Gene Set Enrichment Analysis.
BTLA	B and T-lymphocyte attenuator.
